# Age-Dependent Immune Events during HBV Infection from Birth to Adulthood: An Alternative Interpretation

**DOI:** 10.3389/fimmu.2014.00441

**Published:** 2014-09-23

**Authors:** Antonio Bertoletti, Michelle Hong

**Affiliations:** ^1^Emerging Infectious Diseases (EID) Program, Duke-NUS Graduate Medical School, Singapore; ^2^Viral Hepatitis Laboratory, Singapore Institute for Clinical Sciences, Agency of Science Technology and Research (A*STAR), Singapore

**Keywords:** HBV infection, vertical transmission, immune tolerance, liver inflammation, antiviral immunity

## Abstract

Immune responses change during the life of an individual. While this concept has been well accepted for adaptive immunity, only recently it is becoming clear that the innate immune responses also acquire distinct features in different phases of life. We believe that this concept can offer a different interpretation of the pathological manifestations that can be observed in HBV-infected subjects during the patient’s life. Here, we will review the age-related immunopathological features of HBV infection and discuss how the different virological and clinical manifestations might be linked to the developmental pathway of the immune system from newborns to adults. We will discuss how the age of patients can affect the degree of inflammatory responses, but not the levels of antiviral specific immunity. We then propose that the different clinical manifestations occurring during the natural history of HBV infection are related to the host ability to trigger an inflammatory immune response.

Hepatitis B virus is a hepatotropic, non-cytopathic, DNA virus that chronically infects about 300 million people worldwide. It causes acute or chronic liver diseases characterized by different levels of liver inflammation and viral replication. An acute HBV infection can be resolved without clinical symptoms or with an inflammatory disease of the liver (acute hepatitis). Similarly, persistent HBV infection can cause minimal pathological manifestations or trigger a chronic liver inflammation that develops into liver cirrhosis or cancer (1). These clinical and virological profiles do not only vary among individuals in relation to their genetic background, dose, or route of infection but they are also present within the same individual in relation to his age.

HBV chronic infection is, particularly in Asia, the result of virus transmission from HBV^+^ mothers to their infants (1). HBV infection at birth is characterized by an apparent benign phase of disease with high HBV replication levels and absence of markers of liver inflammation [i.e., normal alanine aminotransferase or aminotransferases (ALT) levels; an enzyme released by dying hepatocytes] during childhood. This phase, defined as immune tolerant, is followed by a disease phase in adulthood characterized by liver inflammatory events and fluctuations in ALT levels and viral load, known as the immune active/clearance phase (Figure 1) (1, 2). The favored interpretation of these different virological and clinical patterns is that during childhood there is a phase of immunotolerance, i.e., an absence of antiviral specific immunity that is lost at the start of adulthood when the host immune system actively “combat” HBV infection (2).

**Figure 1:**
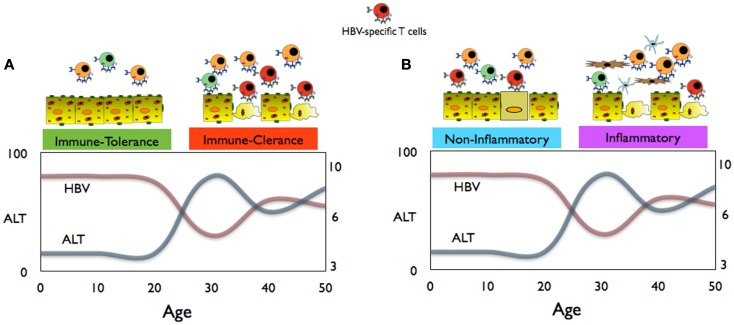
**(A)** Conventional schematic representation of immune events, HBV replication, and aminotransferases (ALT) levels in children and adults during chronic HBV infection. **(B)** Proposed interpretation of the immune events during the non-inflammatory and inflammatory phases of CHB infection.

Here, we will review the data that dispute this scenario and propose an alternative interpretation of these immunopathological events based on the new understanding of the age-related immunological changes.

## HBV Vertical Infection: Does it Induce Immune Tolerance?

The influence of age on HBV infection starts at birth. Take, for example, acute hepatitis B infection. In infants or children, HBV infection rarely causes acute hepatitis (2), while in adults, acute hepatitis B infection is common and is almost invariably associated with HBV control due to the induction of an efficient HBV-specific T- and B-cell response (3). Vertical (mother-to-child) infection in infants often leads to chronic diseases (in 90% of infection) and it is thought that the establishment of a persistent infection is due to immaturity of the neonatal immune system and/or the induction of “immunotolerance,” i.e., the inability to induce a virus-specific immune response (2).

This infant’s inability to mount HBV-specific T- and B-cell responses is supported mainly by data from animal models (i.e., HBV transgenic mice) that showed the presence of immunological defects, which impair HBV-specific T- and B-cell priming in neonatal animals (4–6). These data are methodologically robust. Nevertheless, their significance in relation to HBV pathogenesis can be questionable, since in HBV transgenic mice, HBV does not infect the mouse hepatocytes but viruses are produced through the introduction of HBV–DNA in the mouse genome under the control of hepatocyte-specific promoters (7, 8). As such, the possibility that HBV infection might induce priming of adaptive immunity or activation of innate immunity is excluded “*a priori*.”

The concept that neonates have a “defective” immune system is also changing and mounting evidences show that the neonatal immune responses defy such simple categorization. Indeed, recent studies have demonstrated that the immune effectors as well as regulatory responses are already in place during early fetal life (9, 10). Furthermore, newborns have been shown to mount a virus-specific T cell response toward viral infections in early life (11–13). Besides, exposure of the newborn immune system toward microbes at birth can also modulate the maturation status of the newborn infant. Epidemiological observations and experimental data have shown that bacterial colonization and/or immunization can protect infants against unrelated pathogen infection by inducing an increased functional efficiency of their innate immune system, a process termed “trained immunity” (14). All these earlier reports show that the newborn infant’s immune system is not “defective” *per se*; rather, we believe that it is less prone to trigger a full blown pro-inflammatory reaction, likely as an evolutionary adaptation to avoid undesirable immune reactions *in utero* (15).

Therefore, the hypothesis that vertical HBV infection in infants leads to the induction of immunotolerance remains controversial. Even though the immunological data both during and after natural vertical HBV infection are limited, there are several epidemiological and experimental evidences that contradict the immunotolerance hypothesis during vertical HBV infection. For example, it has been reported that dendritic cell functionality is normal or minimally altered in neonates of HBV^+^ mothers (16, 17) and the detection of T cells specific for HBV in two independent studies performed in HBsAg^−^ children born from HBV^+^ mothers (18, 19) show that neonates exposed to HBV do not necessarily develop a defect in T cell priming but can mount a HBV-specific T cell response. The increase detection of HBV quasispecies in immunotolerant HBV^+^ children is compatible with the presence of an immune pressure (20) and not with tolerance. Similarly, the efficacy of HBV vaccination in children born to HBV^+^ mothers (21, 22) and the better outcome of standard treatment detected in HBV^+^ children (23, 24) are at odds with the general idea of immunotolerance in HBV-infected children. Similarly, the “immunotolerance” hypothesis is difficult to reconcile with epidemiological evidences that HBV-related fulminant hepatitis is more frequent in children of <1 year of age compared to older subjects (25) or with the observations obtained from malaria–HBV co-infected young patients in whom reduced parasitemia (26) and increased incidences of cerebral malaria (27), a Th1-mediated malaria complication, have been reported. This latter observation is perhaps more in line with the possibility of an alternative relationship between HBV and humans during early life.

We have started to investigate the immunological parameters in the cord blood of newborns of HBV^+^ mothers. Characterization of these samples is complex since it is difficult to understand due to ethical considerations whether neonates are infected by HBV (i.e., HBV replication in the hepatocytes) or are only exposed to it (i.e., HBV is present in the circulation but is not able to establish an infection in hepatocytes). Nevertheless, our preliminary data on cytokine analysis of the sera from neonates born to HBV^+^ mothers reveal a cytokine signature compatible with a Th1-like response (i.e., high levels of IL-12p40 and low levels of Th2/suppressive cytokines IL-4, IL-5, IL-13, and IL-10) and a reduced pro-inflammatory cytokine profile (i.e., lower levels of IL-1b and IL-6) (manuscript in preparation). This Th1 cytokine signature is more suggestive of a possible symbiotic relationship between HBV and humans during early life, similar to the one demonstrated in murine model of persistent herpesvirus infection (28), than to the induction of a tolerogenic response. More data need to be gathered to better understand the impact of vertical HBV infection on humans during the early stages of life.

## Immunological Parameters in the “Immune Tolerance” Phase of HBV Infection

The existence of an immunotolerance phase of HBV infection during childhood is supported by clinical and virological manifestations. Since HBV is not directly cytopathic and HBV-specific CD8 T cells control virus spread by killing the infected hepatocytes (29), it is logical to think that the presence/absence of altered ALT levels correlates with the presence/absence of HBV-specific T cells (Figure 1A). Therefore, normality of liver enzymes, detectable in the majority of HBV-infected children, has been interpreted as an indication of a lack of HBV-specific T cell response, while ALT alterations, more likely to occur during adulthood, are interpreted as an “awakening” of HBV-specific immunity.

In reality, ALT measurement cannot be used as a reliable surrogate of a virus-specific T cell response. Work in adenovirus-infected mice (30) confirmed that T cell immunity against hepatocytes could take place without serum ALT elevation. Adoptive transfer of HBV-specific T cells can cause substantial inhibition of HBV replication without ALT increase through cytokine-mediated non-cytopathic virus control (31) and HBV-specific T cells quantification in blood and liver is not proportional to ALT levels (32, 33). Robust liver inflammatory events causing ALT elevations are instead, demonstrated in adult mice and in patients, associated with a liver infiltrate consisting of granulocytes, monocytes, and non-antigen-specific T cells (32, 34, 35).

A direct demonstration that the “immunotolerance” phase is not associated with an absence of virus-specific T cell immunity came directly from our recent study of chronic hepatitis B (CHB)-infected adolescents. Despite having a clinical and virological profile labeled as “immunotolerant,” these adolescents displayed a perfectly normal Th1 T cell response and harbored HBV-specific T cells that were actually functionally better than the ones detected in adult CHB patients in the “immune clearance” phase (36).

## Age-Dependent Changes in Immunity and Inflammatory Events during Viral Infections

Innate immune responses are not identical during the life of an individual. TLR-mediated immune function has been shown to change in different periods of life (37). The production of anti-inflammatory cytokine (e.g., IL-10) is high in preterm infants, and it progressively declines over the first years of life but is superior in children versus adults. In contrast, the production of pro-inflammatory cytokines (e.g., IL-1b, TNF-alpha) steadily increases during early life (37) until it reaches the state of chronic low-grade systemic inflammation called “inflammaging” present in elderly subjects (38).

Consequently, T cell responses also shift from a Th2/Treg type response in newborns to a more Th1 response in children/adults (15) and with a progressive increase in effector memory T cell pools. These effector memory T cells can respond efficiently to a cognate infection and are also sensitive to cytokine-mediated activation (39). The question then arises: can these age-dependent changes in immune functions explain the different virological and inflammatory patterns during the natural history of HBV infection?

**Figure 2:**
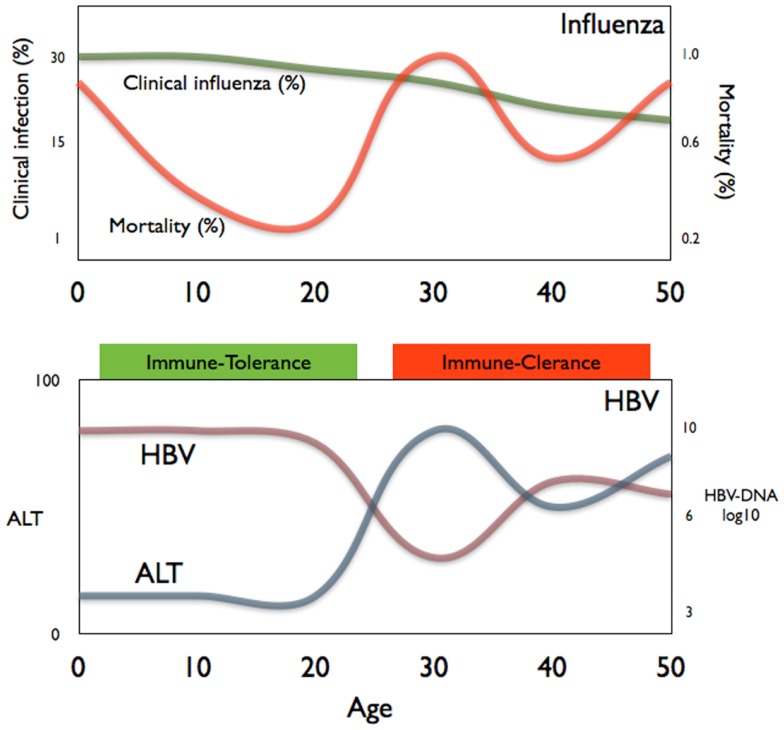
**A comparison of the age-related mortality of influenza (top panel) and the age-related phases of chronic HBV infection (bottom panel)**.

Interestingly, the evidence of the impact of age on pathological processes can also be observed in other infections. Influenza virus infection, for example, can cause death due to an increased inflammatory response in the lung predisposing to bacterial infection (40) in some but not all individuals. Figure 2 shows the data on the incidence of both clinical influenza and pneumonia mortality during the 1918 influenza pandemic in relation to age (40). It is evident that children, despite experiencing a higher rate of clinical influenza, had a much lower mortality rate than young adults. This phenomenon has also been observed in other infections (41). The immunological mechanisms underlying these different outcomes have not been experimentally demonstrated but the common trend is that inflammatory events are less severe in children than in adults. It is certainly intriguing that the “immunotolerant and the active immune phases” of chronic HBV infection mirrors the different age-related mortality seen in the 1918 influenza pandemic (Figure 2).

## Immunological Events during HBV Infection: An Alternative Hypothesis

Based on the epidemiological, clinical, and experimental data summarized above, we propose an alternative interpretation of the immune events occurring during natural HBV infection:

(a) HBV transmission at birth is not exclusively followed by persistent infection and characterized by an inability to mount a HBV-specific adaptive immunity. Newborns of HBV^+^ mothers can mount a HBV-specific immune response following vaccination (22), and the detection of T cells specific for the HBV core and polymerase antigen (antigens not present in the HBV prophylactic vaccine) (18, 19) shows that HBV exposure *in utero* can prime virus-specific T cells. Further studies are needed to define the mechanisms/circumstances underlying this induction. However, the frequent detection of HBV-specific memory T cells in young, exposed, but uninfected subjects (42) imply that the induction of a specific adaptive immunity after HBV contact at birth or during childhood is not a rare event. However, with the exceptions of very few cases of fulminant hepatitis present at birth (25), HBV-specific adaptive immunity seems to occur in children without symptoms of acute hepatitis. Whether this different clinical phenotype is the result of a quantitative/qualitative defect in T cell response or a reduced ability of children to mount a pro-inflammatory response needs to be further evaluated.
(b) The state of high HBV replication and low ALT levels present in young CHB patients should be termed the “non-inflammatory” phase of HBV infection (Figure 1B). Data obtained from young CHB patients (age between 15 and 30 years old) show that, at least in this period, the absence of serological markers of liver inflammation is not associated with an immune profile of general or HBV-specific T cell tolerance (36). Furthermore, clonal hepatocyte repopulation, an indirect measurement of targeted killing of HBV-infected hepatocytes, has been detected in immunotolerant patients (P. Kennedy and Bill Mason, personal communications). The hypothesis is that in children, HBV-specific CD8 T cells might try to contain the HBV infection like in adults but due to the reduced pro-inflammatory cytokine milieu and the limited pool of effector/memory T cells, they do not trigger the non-specific recruitment of inflammatory cells to the liver.
(c) The immune clearance phase of chronic hepatitis, characterized by altered levels of ALT, fluctuations in the HBV replication levels, and histological signs of liver inflammation, may be better defined as the “inflammatory” phase of CHB (Figure 1B). The adult’s propensity to mount pro-inflammatory immune reactions might explain the clinical, immunological, and virological profiles of HBV infection in adults. Liver inflammation can be triggered by HBV-specific CD8 T cells, but it is unlikely a direct effect since the extent of liver inflammation is not proportional to the absolute quantity of HBV-specific CD8 T cells. In adult patients, HBV-specific T cell response is not proportional to liver inflammation (32). Similarly, episodes of hepatic flares (heightened liver inflammation) are observed without any detectable increase of circulating HBV-specific T cells (43) but are instead associated with increased level of chemokines (43) and NK cell activation (44). Changes in liver inflammatory environment might tune the sensitivity of T cells to antigens (45) and this might indirectly increase HBV-specific T cell activation. Alternatively, liver inflammatory events might derive directly from pro-inflammatory reactions trigger by different sources, e.g., bacterial products as shown in a model of hepatic steatosis (46). Changes in the intra-hepatic pro-inflammatory environment may explain the fluctuations in HBV replication. IL-1beta and TNF-alpha, the classical pro-inflammatory cytokines, have inhibitory effects on HBV (47, 48) through the activation of cytidine deaminases, enzymes that are highly activated in advanced liver pathologies (49).

There are clinical implications on these alternative interpretations. Firstly, if young CHB patients can mount HBV-specific immune responses without inducing a full blown pro-inflammatory reaction, therapies designed to boost HBV-specific immunity (e.g., vaccine therapy, check points inhibitors) (50) are more likely to succeed and will be less damaging in these young patients. Secondly, if we start to evaluate and study CHB infection in adults in the context of an inflammatory disease rather than a viral induced disease, anti-inflammatory therapies designed to inhibit the liver inflammatory events may prove to be important in controlling HBV infection. The superior efficacy of the anti-platelet therapy in blocking the development of hepatocellular carcinoma in HBV transgenic mice is one such example (51, 52).

## Concluding Remarks

Epidemiological and experimental evidences raised doubts that the natural progression of CHB can be dictated by a simple quantitative difference in adaptive immunity. The classic view of HBV vertical transmission inevitably causing a phase of HBV-specific immune tolerance, which is lost during adulthood, is not tenable to our increased understanding of the effect of age on the immune response and on the HBV-specific immune events.

We are aware that more experimental data need to be gathered to support the alternative scenarios that we have proposed. Nevertheless, we and others (53) believe that the different clinical phenotypes of HBV infection in children and adults should no longer be described based on (1) the mechanisms that have been exclusively determined in experimental mouse models that do not establish persistent HBV infection and (2) the clinical observations on the circulating levels of HBV and ALT.

Having said that, additional data need to be gathered especially during the early phases of HBV infection, i.e., from HBV-exposed newborn infants and CHB-infected children, in whom limited studies have been performed. Moving forward, it is of our interest to test whether HBV^+^ children harbor HBV-specific immunity and whether such responses trigger intra-hepatic inflammatory events only in adults. We are also aware of the potential roles of many other components of the immune response that needs to be analyzed; one example is NK cells that are present abundantly in the liver and might have both antiviral and regulatory effects in HBV infection (54).

At the moment, there are still many gaps in the knowledge of HBV infection and we hope that this opinion article might inspire new avenues of research that can better define the immunopathological processes of this infectious disease that is affecting a considerable part of human population.

## Conflict of Interest Statement

The authors declare that the research was conducted in the absence of any commercial or financial relationships that could be construed as a potential conflict of interest.
